# Mitophagy Failure in Fibroblasts and iPSC-Derived Neurons of Alzheimer’s Disease-Associated Presenilin 1 Mutation

**DOI:** 10.3389/fnmol.2017.00291

**Published:** 2017-09-14

**Authors:** Patricia Martín-Maestro, Ricardo Gargini, Andrew A. Sproul, Esther García, Luis C. Antón, Scott Noggle, Ottavio Arancio, Jesús Avila, Vega García-Escudero

**Affiliations:** ^1^Departamento de Neurobiología, Centro de Biología Molecular “Severo Ochoa” (UAM-CSIC) Madrid, Spain; ^2^Centro de Investigación Biomédica en Red de Enfermedades Neurodegenerativas (CIBERNED) Madrid, Spain; ^3^Brain and Mind Research Institute, Weill Cornell Medical College, Cornell University New York, NY, United States; ^4^Department of Pathology & Cell Biology and the Taub Institute for Research on Alzheimer’s Disease and the Aging Brain, Columbia University New York, NY, United States; ^5^The New York Stem Cell Foundation New York, NY, United States; ^6^Departamento de Anatomía, Histología y Neurociencia, Facultad de Medicina, Universidad Autónoma de Madrid Madrid, Spain

**Keywords:** Alzheimer’s disease, presenilin 1 mutation, mitophagy, fibroblasts, iPSC derived neurons

## Abstract

Familial Alzheimer’s disease (FAD) is clearly related with the accumulation of amyloid-beta (Aβ) and its deleterious effect on mitochondrial function is well established. Anomalies in autophagy have also been described in these patients. In the present work, functional analyses have been performed to study mitochondrial recycling process in patient-derived fibroblasts and neurons from induced pluripotent stem cells harboring the presenilin 1 mutation A246E. Mitophagy impairment was observed due to a diminished autophagy degradation phase associated with lysosomal anomalies, thus causing the accumulation of dysfunctional mitochondria labeled by Parkin RBR E3 ubiquitin protein ligase (PARK2). The failure of mitochondrial recycling by autophagy was enhanced in the patient-derived neuronal model. Our previous studies have demonstrated similar mitophagy impairment in sporadic Alzheimer’s disease (AD); therefore, our data indicate that mitophagy deficiency should be considered a common nexus between familial and sporadic cases of the disease.

## Introduction

Alzheimer’s disease (AD) is a common and devastating dementia that is pathologically defined by the accumulation of extracellular amyloid beta (Aβ)-containing amyloid plaques and intraneuronal hyperphosphorylated Tau protein aggregates associated with neuronal loss in the cerebral cortex. Over 200 mutations in the amyloid precursor protein (APP) or the presenilin 1 and 2 genes (*PSEN1/2*) can cause dominantly inherited, early-onset forms of AD, termed Familial Alzheimer’s disease (FAD; Dinnage et al., 2012). Mutations in the *PSEN1* gene, located on chromosome 14, occur most frequently in FAD. Defective presenilin 1 interferes with the function of the γ-secretase complex, which alters the processing of the APP and leads to the overproduction of a longer, toxic version of Aβ peptide (Aβ42; Borchelt et al., 1996). Generation of toxic Aβ peptide and the formation of amyloid plaques likely lead to the death of neurons and synaptic dysfunction.

The *PSEN1* A246E mutation (FAD1 family) was originally reported in 1995 in conjunction with the cloning of the *PSEN1* gene (Sherrington et al., 1995). It was detected in a Canadian family of Anglo Saxon-Celtic origin. The pattern of transmission is consistent with autosomal-dominant inheritance, and genetic analysis confirmed that the mutation segregated with disease. Moreover, *PSEN1* A246E is reported to induce elevation of Aβ42 in human plasma, patient-derived fibroblasts, forced-expressed cells and showing strong toxicity in mice (Borchelt et al., 1996). Besides the generation of Aβ, little is known about PSEN1 implication in mitochondrial dysfunction and oxidative damage.

Mitophagy is the selective degradation of mitochondria by autophagy. It promotes turnover of mitochondria and prevents accumulation of dysfunctional mitochondria which can lead to cellular degeneration (Lemasters, 2005). PARK2-dependent mitophagy is one of the best studied mechanisms for mitophagy in mammalian cells (Fedorowicz et al., 2014) where PARK2 RBR E3 ubiquitin protein ligase (PARK2), a cytosolic E3 ubiquitin l ligase, translocates to depolarized mitochondria and initiates their degradation via autophagy. It has been described that PARK2 is recruited to the mitochondria by a PTEN-induced putative kinase 1 (PINK1)-dependent mechanism (Vives-Bauza et al., 2010). After mitochondrial damage, PINK1 is stabilized in the mitochondrial membrane as a full length (FL) isoform that recruits PARK2 (Narendra et al., 2010). PARK2 mediates the ubiquitination of several mitochondrial proteins like VDAC1 (Geisler et al., 2010) and mitofusins (Gegg et al., 2010). This induces the recruitment of autophagy adapter proteins, such as sequestosome 1 (SQSTM1/p62) which interacts with MAP1LC3/microtubule-associated protein 1 light chain 3 (LC3) mediating the cargo engulfment into autophagosomes for final recycling by fusion with lysosomes (Bjørkøy et al., 2005). On the other hand, it has been recently demonstrated PINK1 may also play an inhibitory role in mitophagy process (Fedorowicz et al., 2014). The main cleaved product of PINK1 (Δ1 fragment), is able to physically bind PARK2 in the cytosol inhibiting its translocation to the mitochondria, therefore, impairing the elimination of damaged mitochondria.

The AD-related mitophagy was previously reported by the accumulation of autophagic vesicles (AV) in pyramidal neurons from AD patients suggesting a mitophagy alteration (Moreira et al., 2007a,b). Previous study defined an essential role for PSEN1 in the maturation and trafficking of the v-ATPase responsible for lysosome acidification which is essential for the normal turnover of proteins and organelles by autophagy (Lee et al., 2010) although, its involvement in mitophagy was not reported. Moreover, it has been described that *PSEN1* deficient cells exhibited decreased Ca^2+^ storage in degradative organelles besides a significantly decreased lysosomal Ca^2+^ release, demonstrating a cell type-independent function for PSEN1 in lysosomal Ca^2+^ homeostasis promoting the consequent defect in lysosomal fusion and protein clearance (Coen et al., 2012).

Recent advances in iPSC technology grant access to unique human samples and enable the generation of disease- and patient-specific cell lines. Terminally differentiated cell types, such as neurons, derived from iPSC lines are extremely useful for understanding the patho-physiological mechanisms of AD (Sproul, 2015). Thus, we evaluated the mitochondrial recycling process by autophagy in two different human cell models of FAD-associated *PSEN1* A246E mutation: unmodified skin fibroblasts and iPSC-derived neurons. Patients-derived skin fibroblasts harboring FAD1 mutation demonstrated a defect in degradation phase of autophagy correlating with lysosomal anomalies, leading to accumulation of dysfunctional mitochondria consistent with mitophagy impairment. We confirmed these results in neurons derived from AD patients’ iPSC harboring the same mutation, therefore demonstrating an exacerbation of mitophagy failure leading to increased mitochondrial accumulation.

## Materials and Methods

### Primary Cells and Culture Conditions

Primary skin fibroblasts from AD patients and their correspondent control sex and age-matched samples were obtained from Coriell Institute for Medical Research (New Jersey, USA). iPSC lines were generated by Andrew Sproul and Scott Noggle from New York Stem Cell Foundation from skin fibroblast cell lines (Sproul et al., 2014). See Table 1 for details about age, sex and disease state of the correspondent patients whose cells were used.

**Table 1:** Core set of skin fibroblasts lines.

Line	Age/Sex	Clinical diagnosis
AG04148	56/M	Non-affected
AG12988	56/F	Non-affected
6842 A (iPSC)	75/M	Non-affected
AG06840	56/M	Moderate dementia (*PSEN1* A246E)
AG06848	56/F	Severe dementia (*PSEN1* A246E)
7671 C (iPSC)	44/M	Moderate dementia (*PSEN1* A246E)

*Coriell Institute for Medical Research*.

Human fibroblasts were cultured in Dulbecco’s modified Eagle’s medium (DMEM) supplemented with 10% (v/v) heat-inactivated Fetal Bovine Serum (FBS), 2 mM glutamine, 10 U/ml penicillin, 10 μg/ml streptomycin, in 5% CO_2_ in a humid incubator at 37°C. The use of fibroblasts has been restricted to a maximum of 10 cell passages to avoid replicative senescence and cultures were always maintained below confluence.

Undifferentiated iPSCs were kept onto Cultrex (Trevigen, 3432-005-01)-coated dishes and grown in feeder-free maintenance basal medium for hESCs and hiPSCs mTesR1 (StemCell Technologies, 05851) supplemented with mTeSR1 5× Supplement (StemCell Technologies, 05852) and penicillin-streptomycin (100 U/mL–0.1 mg/mL) in 5% CO_2_ in a humid incubator at 37°C.

For neuronal cortical differentiation, iPSC colonies were dissociated into single cells by washing with PBS and adding 1 ml Accutase (Life Technologies, A1110501) and then plated onto Cultrex-coated dishes in mTesR1 medium containing 10 μM ROCK inhibitor (Y-27632, Stemgent, 04-0012). Cells were plated at a density of 3 × 10^5^ cells/well in six well-plate and allowed to recover for 3 days to reach near confluence. Cells were neuronally differentiated with dual-smad inhibition from days 0 to 9 in custom TesR1 (5× supplement mTesR1 and penicillin-streptomycin (100 U/mL–0.1 mg/mL) using 10 μM SB431542 (Stemgent, 04-0010) and 250 nM LDN193189 (Stemgent, 04-0074). Cell were split with Accutase on day 10 and plated at a density of 2 × 10^5^ cells per six well-plate on 100 μg/mL Poly-L-Ornithine (Sigma-Aldrich, P3655) and 3 μg/mL laminin (Sigma-Aldrich, L2020) plates. Monolayer neuronal differentiation was carried out in Neurobasal (Life Technologies, 21103049) with B27 supplement (Life Technologies, 17504044) and cells were fed every 2–3 days until analyzed. At day 30, neuronal differentiation media was supplemented with 20 ng/ml BDNF (R&D system, 2837).

Human subjects research at the New York Stem Cell Foundation was performed in accordance with applicable federal and state regulations, as well as with guidelines established by the National Institutes of Health (NIH), National Academy of Sciences (NAS) and International Society for Stem Cell Research (ISSCR), and was also compliant with standards outlined in the Health Insurance Portability and Accountability Act (HIPAA) and by the Office for Human Research Protections (OHRP). The study was approved by the Sub-Committee on Bioethics of Consejo Superior de Investigaciones Científicas (CSIC).

### Antibodies

The primary antibodies are shown in Table 2. The secondary antibodies for Western blot studies were horseradish peroxidase–conjugated anti-rabbit, anti-mouse or anti-goat IgGs (P0448 and P0161 or P0160, DAKO) and for immunofluorescence were anti-mouse, anti-rabbit or anti-guinea pig IgGs alexa-488, -555 or -647 labeled (Molecular Probes, Millipore).

**Table 2:** Antibodies used in the study.

Antibody	Reference
APP	A8717, Sigma-Aldrich
BECN1	sc-11427, Santa Cruz
Cathepsin B	sc-6493, Santa Cruz
Calbindin	CB-38a, Swant
EEA1	610457 BD Biosciences
GAPDH	ab8245, Abcam
LAMP1	611042, BD Bioscience
LAMP2	sc-18822, Santa Cruz
LC3	B7931, Sigma-Aldrich for WB
LC3	M152-3, MBL for immunostaining
MAP2	M4403, Sigma-Aldrich
Nanog	4903S, Cell Signaling
NeuN	ABN78, Millipore
Oct4	09-0023, Stemgent
PARK2	sc-32282, Santa Cruz
PINK1	BC100-494, Novus
p62	610832, BD Bioscience
Rab5	3547, Cell Signaling
Rab7	9367, Cell Signaling
SSEA4	ab16287, Abcam
Sox2	09-0024, Stemgent
Tau1	MAB3420, Millipore
Tau5	577801, Calbiochem
Tra1-60	MAB4360, Millipore
Tra1-81	MAB4381, Millipore
TOMM20	sc-11415, Santa Cruz
vGlut1	AB5905, Millipore
β-actin	A5441, Sigma-Aldrich
β-tubulin	T4026, Sigma-Aldrich

### Western Blot Analysis

The cells and tissue samples were homogenized in lysis buffer (50 mM pH 7.5 HCl-Tris, 300 mM NaCl, 0.5% SDS and 1% Triton X-100) and incubated 15 min at 95°C. Protein concentration of the extracts was measured using the Dc protein assay kit (500-0111, Bio-Rad). Equal amounts of total protein extract from control and FAD cells were resolved by SDS-PAGE and then transferred to nitrocellulose (G9917809, Amersham, Germany) or PVDF (IPVH00010, Merk Millipore, Cork, Ireland) membranes. Western blot and immunoreactive proteins were developed using an enhanced chemiluminescence detection kit (NEL105001EA, Perkin Elmer) following instructions of the supplier. Quantification was performed by densitometry of the obtained bands in each lane with respect to the correspondent housekeeping protein in each experiment (Quantity One software, Bio-Rad). When molecular weights were different enough, sometimes the same membrane was re-used to detect several proteins. In this way, the housekeeping protein pattern might appear in different western blots for different proteins. When indicated, data was normalized with respect to the values obtained from each correspondent age-matched control sample.

### Autophagy Flux Study

Fibroblasts and iPSC-derived neurons were treated with 20 μM carbonyl cyanide m-chlorophenylhydrazone (CCCP; C2759, Sigma-Aldrich) for 24 h followed by an additional treatment of PBS or NH_4_Cl (15 mM) for 6 h in the presence of CCCP. After the treatment, cells were lysed in Western blot buffer and immunodetection of autophagy-involved proteins was performed as described. Quantification of autophagic synthesis was represented as the ratio between the values of the cells treated with CCCP and NH_4_Cl with respect to the condition without CCCP but maintaining NH_4_Cl treatment. Quantification of autophagic degradation ratio was obtained by the relation between the values of the cells treated with CCCP and NH_4_Cl and the ones without NH_4_Cl but maintaining CCCP treatment according to autophagy standard guidelines (Klionsky et al., 2016). See Supplementary Figure S1 for scheme of the analysis based on previous representation (Rubinsztein et al., 2009).

### Immunocytochemistry

Fibroblasts and differentiated neurons were grown on sterile glass coverslips, treated as described for each experiment, followed by washing with PBS and fixing with 4% paraformaldehyde in PBS for 30 min or 10 min for fibroblasts or neurons respectively at room temperature. After blocking with PBS containing 1% BSA, permeabilization with 0.1% Triton X-100/PBS and Glycine 1 M for 30 min, cells were washed with PBS and stained by indirect immunofluorescence using the antibodies described before. When indicated, phalloidin (A-12379, Life Techonolgies) was added at 1:200 during the second antibody incubation. Samples were mounted with Prolong Gold Antifade (P-36930, Life Technologies) and randomly chosen field images were obtained in an Invert Confocal LSM510 (Zeiss, Oberkochen, Germany) fluorescence microscope. iPSC colonies were fixed with 4% for 10 min and blocked with blocking buffer (PBS with 0.25% TritonX-100, 2% bovine serum albumin, and 1% sodium azide) for 30 min. Cells were then stained with primary antibody diluted according to manufacturer recommendation in blocking buffer overnight at 4°C. Cells were washed with 0.1% Triton X-100/PBS and stained with secondary antibody (1:400 in blocking buffer) for 2 h at room temperature. Cells were washed again with 0.1% Triton X-100/PBS and images were taken on an Olympus IX71 inverted microscope.

### Quantification of Colocalization and Vesicles Average Size

Colocalization analysis was performed with ImageJ software (Bethesda, MD, USA) and every cell was manually delimited according to phalloidin staining. The background of different channels was edited with Subtract Background tool with a rolling ball radius of 30 pixels; and by a threshold intensity, binary images were obtained. The logical operation AND and Subtract of the Image Calculator tool was used to generate an image harboring only overlapping structures of both channels. Colocalization measurement was obtained by quantifying the area occupied by the overlapping elements per cell. At least 200 cells were measured for each cell line. For the endo-lysosomal size analysis, cells were thresholded and the average size was calculated using the particle analyzer of ImageJ software.

### Mitochondrial Content Analysis

Mitochondrial content was measured using an ImageJ macro designed by Dagda et al. (2009) at the University of Pittsburgh. The macro allows one to threshold immunostained mitochondria in cells labeled with a mitochondrial antibody or transiently expressing mitochondrially targeted GFP. It displays measurements of several mitochondrial parameters including total michondrial area. In our case, we used the total area of all mitochondria measured per cell. For quantification of mitochondrial content at least 1000 cells were measured for each cell line.

### Mitochondrial Potential Measurement

Cells were treated with CCCP (20 μM) for 7 h and, in the reversible condition, CCCP was removed and the medium was replaced for DMEM 10% FBS during the last 60 min. Then, fibroblasts were incubated with 50 nM DiOC6(3) (D273, Molecular Probes; Sauvat et al., 2015) for 30 min at 37°C and analyzed by flow cytometry (FACSCalibur, BD Biosciences, San Jose, CA, USA). Mitochondrial membrane potential for each condition was represented as the percentage of the fluorescence intensity with respect to fluorescence intensity exhibited when these cells remained untreated.

### Lysosomal Function Study

Cells were treated with 100 nM bafilomycin A1 (B1793, Sigma-Aldrich) for 2 h or remain untreated. After that, cells were collected and treated with 500 nM LysoTracker Red DND-99 (L-7528, Molecular Probes, Life Technologies) for 20 min. Then, cells were analyzed by flow cytometry (FACSCalibur, BD Biosciences, San Jose, CA, USA). Relative LysoTracker level represents the ratio of the FL-2 fluorescence intensity of the untreated condition and the bafilomycin treated one.

### Maturation of Mitophagic Vesicles Measurement

The mCherry-EGFP-LC3B construct was obtained from Dr. Terje Johansen (Institute of Medical Biology, University of Tromsø, Norway; Pankiv et al., 2007). Briefly, pDest-mCherry-EGFP-LC3B was digested with XbaI and NheI, and mCherry-EGFP-LC3B fragment was subcloned into pLenti CMVTO Empty Neo (Addgene plasmid #17485) digested with XbaI. Orientation in the final vector pLenti-mCherry-EGFP-LC3B was checked by digestion with XbaI and XhoI that releases the construct only when inserted in the right orientation.

Lentiviral particles were packaged as previously described (Martín-Maestro et al., 2016) by co-transfection of HEK293T cells with 10 μg of pLenti-mCherry-EGFP-LC3B plasmid, 6 μg of the packaging plasmid pCMVdR8.74 (Addgene plasmid 22036) and 5 μg of the VSV G envelope protein plasmid pMD2G (Addgene plasmid 12259) using Lipofectamine and Plus reagents following instructions of the supplier (18324 and 11514 respectively, Life Techonologies). Lentiviral titer was calculated by infecting cells with increasing amounts of lentiviral supernatant and the percentage of infected cells was detected by flow cytometry (FACSCalibur, BD Biosciences).

Fibroblasts were infected with lentivirus encoding mCherry-EGFP-LC3B at a multiplicity of infection of 5 infective units/cell. After 72 h, cells were treated with CCCP (20 μM) for 6 h and were subsequently fixed with 4% paraformaldehyde. Immunofluorescence for Translocase of outer mitochondrial membrane 20 homolog (TOMM20) was performed as described in “Immunocytochemistry” Section. Twenty-five randomly chosen field images per condition were obtained in a LSM710 Zeiss confocal microscope (Zeiss) and colocalization was quantified as described in “Quantification of Colocalization and Vesicles Average Size” Section.

### Microarray Analysis

The analysis of specific gene expression profiling by array (Affymetrix Human Gene 1.0 ST Array) of Lysosomal-associated membrane protein 1 (LAMP1), LAMP2, EEA1, TFEB, KPNA2, ATP6V0E1, CTSL1, ARSB, GNS and ONECUT2 of human brain samples classified into control (*n* = 7) vs. FAD caused by *PSEN1* mutation (*n* = 7) was obtained from Antonell study (Antonell et al., 2013). Differences in gene expression between control and FAD were calculated using Student’s *t*-test.

### Statistical Analysis

Graphs represent means and standard deviations of the values obtained from experimental triplicates using one couple of sex and age-matched control and FAD1 samples which are specified in each legend to the figure. When necessary, values represented in the graphs were obtained by normalizing every FAD1 sample data with its correspondent age-matched control sample. Statistical comparison of the data sets was performed by the Student’s *t* test. Two-way analysis of variance (ANOVA) test was performed to examine the differences between experimental factors and their interaction. A *post hoc* Bonferroni test was used when more than two experimental groups were compared. When the distribution of the data was not normal a non-parametric Mann–Whitney U test was used. The differences are given with their corresponding statistical significance or *p* value, which is the probability that the difference occurred merely by chance under the null hypothesis.

## Results

### Autophagy Degradation Impairment in *PSEN1*-FAD Fibroblasts

**Figure 1:**
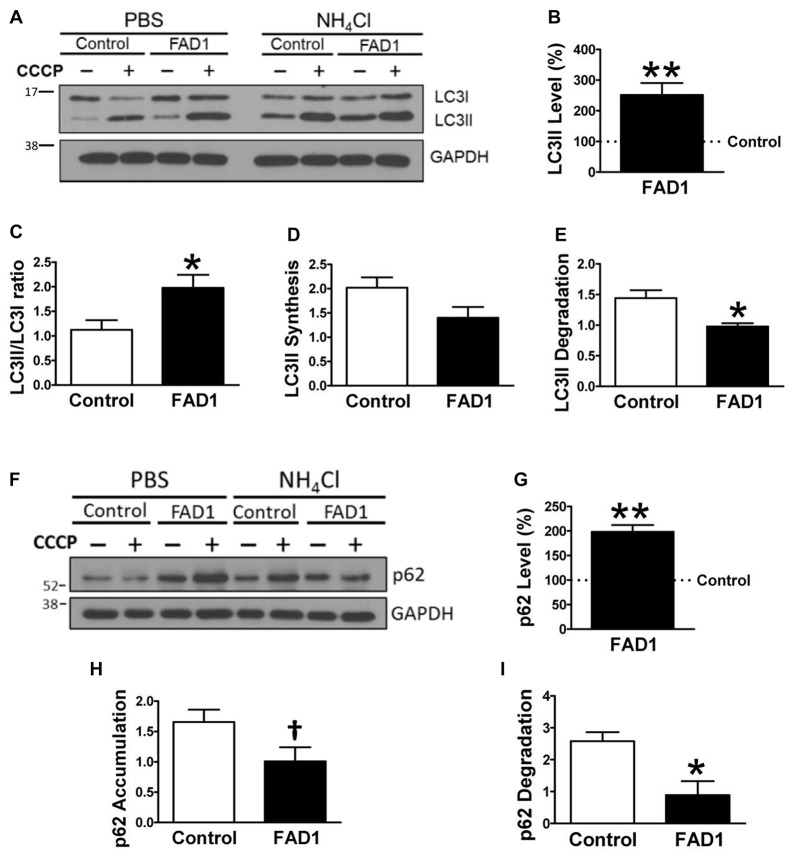
Autophagy degradation phase impairment in Familial Alzheimer’s disease associated to presenilin 1 A246E mutation (FAD1) fibroblasts. **(A)** Representative Western blot of Microtubule-associated protein 1 light chain 3 (LC3) expression for the study of autophagy flux as explained in “Materials and Methods” Section and Supplementary Figure S1, in control and FAD1 fibroblasts treated or not with carbonyl cyanide m-chlorophenylhydrazone (CCCP; 20 μM) in the absence or presence of NH_4_Cl (15 mM). **(B,C)** Quantification of LC3II levels **(B)** and LC3II/LC3I ratio **(C)** in FAD1 cells with respect to the control ones under basal conditions. **(D,E)** Quantification of LC3II synthesis **(D)** and degradation **(E)** ratios as described in “Materials and Methods” Section. **(F,G)** Western blot of p62 expression after the treatment as in **(A)** and quantification of basal levels **(G)**. **(H,I)** Quantification of p62 accumulation **(H)** and degradation **(I)** ratios (*n* = 3 independent experiments using the control/Alzheimer’s disease (AD) fibroblast couple AG04148/AG06840; ^†^*p* < 0.08; *p < 0.05; **p < 0.01).

**Figure 2:**
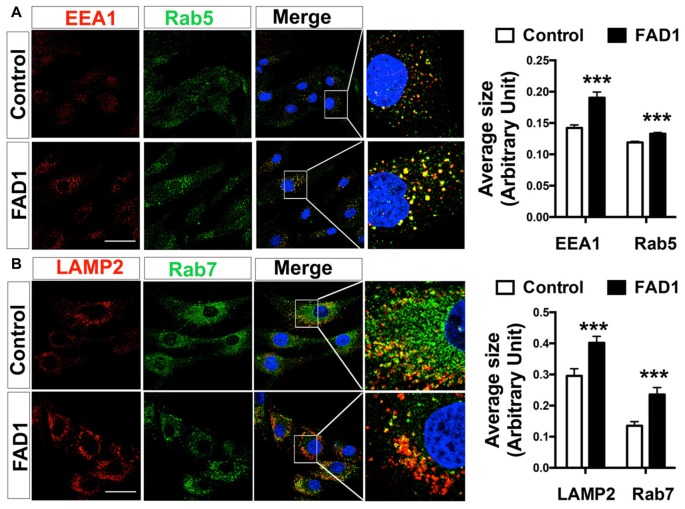
Altered endolysosomal compartment in FAD1 fibroblasts. **(A)** Representative confocal microscopy immunofluorescence images showing EEA1 in red and Rab5 in green of control and FAD1 fibroblasts in basal conditions. Images on the right show enlargement of the squared area. Quantification of average size of early endosomes using each label. **(B)** Similar study as shown in **(A)** with the lysosomal markers Lysosomal-associated membrane protein 2 (LAMP2) in red and Rab7 in green and quantification of the data (Control/AD fibroblast couple used AG04148/AG06840; ***p < 0.001). Scale bar: 40 μm.

To identify whether autophagy may be altered in our *PSEN1*-FAD (FAD1) fibroblasts, we treated the cells with the mitochondrial respiratory chain uncoupler CCCP, which triggers loss of mitochondrial membrane potential. Cells were treated with CCCP for 24 h followed by an additional treatment of NH_4_Cl for 6 h in the presence of CCCP to block the degradation phase of autophagy. We have previously demonstrated that these treatments were not toxic for human fibroblasts (Martín-Maestro et al., 2016). Western blot analysis revealed an increase of LC3II and LC3II/LC3I ratio levels in FAD1 fibroblasts compared to control samples (Figures 1A–C). This may reflect an accumulation of AV in FAD1 cells. According to this, we could observe a diminished autophagy flux where the autophagosome (LC3II) synthesis was slightly decreased but the degradation was drastically reduced in FAD1 samples (Figures 1D,E). These data correlated with the significant increase of LC3II levels indicating that the degradation phase of autophagy was blocked by PSEN1 alteration caused by FAD mutations. To confirm this hypothesis, we examined the turnover of p62, an adaptor protein degraded by autophagy, under the same conditions. We observed that basal levels of p62 were notably elevated in FAD1 cells compared with control samples (Figures 1F,G) as had been previously reported (Lee et al., 2010). Moreover, these cells exhibited decreased p62 accumulation besides a remarkably lower degradation of p62 (Figures 1H,I). In order to assess the generality of our findings we performed a similar study using another pair of control and FAD fibroblasts harboring the same mutation observing similar results (Supplementary Figures S2A–I). Accumulation of LC3II and p62 together with a reduction of degradation phase in both control/FAD1 couples strongly suggests that lysosomal function may be inhibited in FAD1 fibroblasts. To address a possible lysosomal dysfunction, we first studied the levels of lysosomal markers in FAD1 fibroblast such as LAMP1, LAMP2 and Rab7 as well as early endosomal markers like EEA1 and Rab5 in both fibroblasts couples (Supplementary Figure S3). However, we could not find clear differences in the levels of these proteins between FAD1 fibroblasts with respect to controls that could explain lysosomal dysfunction, because only slight increase of some proteins was obtained (Supplementary Figure S3). On the contrary, we could observe a clear increase of the average size of the early endosomes and lysosomes (Figures 2A,B, respectively) which has been previously described in AD patients brain (Cataldo et al., 1997; Yamano et al., 2016). Conversely, the acidification of lysosomes was diminished in FAD1 cells with respect to controls (Supplementary Figure S2J). All these data correlate with previously published results that indicate that PSEN1 function is necessary for the targeting of the v-ATPase V0a1 subunit to lysosomes, their acidification and function (Lee et al., 2015). Therefore, *PSEN1* mutations impair degradative phase of autophagy (Lee et al., 2010; Wolfe et al., 2013).

### FAD1 Fibroblasts Exhibit Misbalance of Mitophagy-Involved Proteins

**Figure 3:**
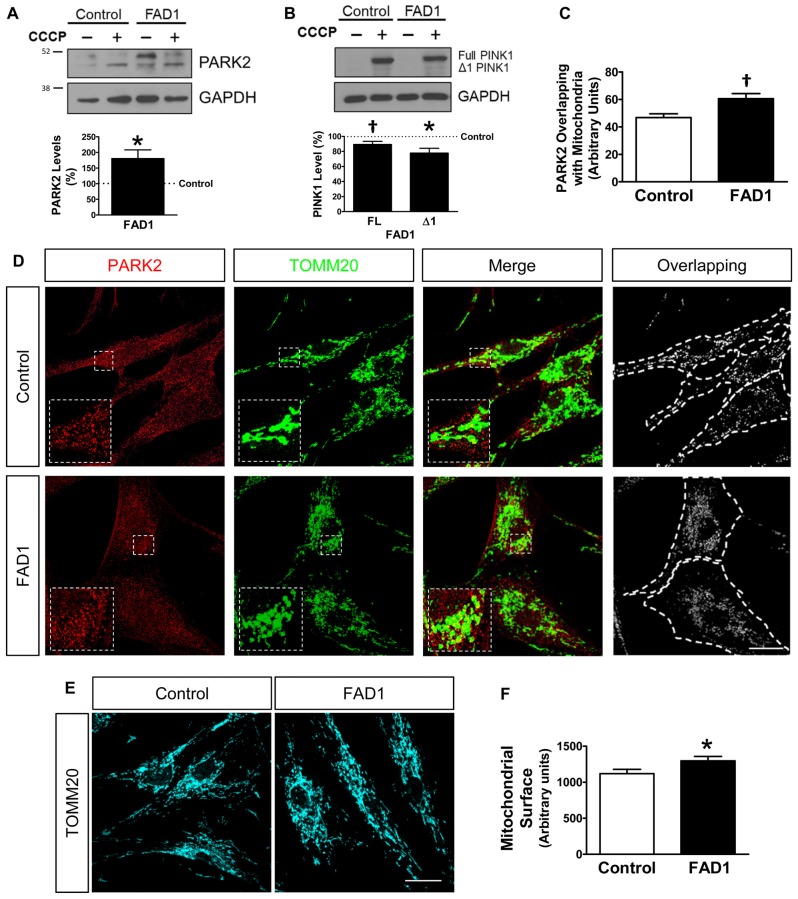
Dysfunctional mitochondrial clearance in FAD1 fibroblasts. **(A)** Representative Western blot of control and FAD1 fibroblasts in the absence or presence of CCCP (20 μM) for 24 h and quantification of Parkin RBR E3 ubiquitin protein ligase (PARK2) levels under basal conditions. **(B)** Representative Western Blot and quantification of full length (FL)-PTEN-induced putative kinase 1 (PINK1) and Δ1-PINK1 after the treatment with CCCP. **(C,D)** Quantification of the colocalization between PARK2 and Translocase of outer mitochondrial membrane 20 homolog (TOMM20) expressed as area occupied by the overlapping elements per cell data **(C)** from representative confocal microscopy immunofluorescence images showing PARK2 in red and TOMM20 as a mitochondrial constitutive marker in green in the same cells treated with CCCP (20 μM) for 1 h **(D)**. Inserts represent a magnification of the same squared representative area. On the right, binary image representing the colocalization of both labels and dotted line delimits cytoplasm of each cell according to phalloidin staining (not shown). **(E,F)** Representative confocal images and quantification of the mitochondrial surface per cell showing TOMM20 in cyan in basal conditions (*n* = 3 independent experiments using the control/AD fibroblast couple AG04148/AG06840; ^†^*p* < 0.08; *p < 0.05). Scale bar: 40 μm.

**Figure 4:**
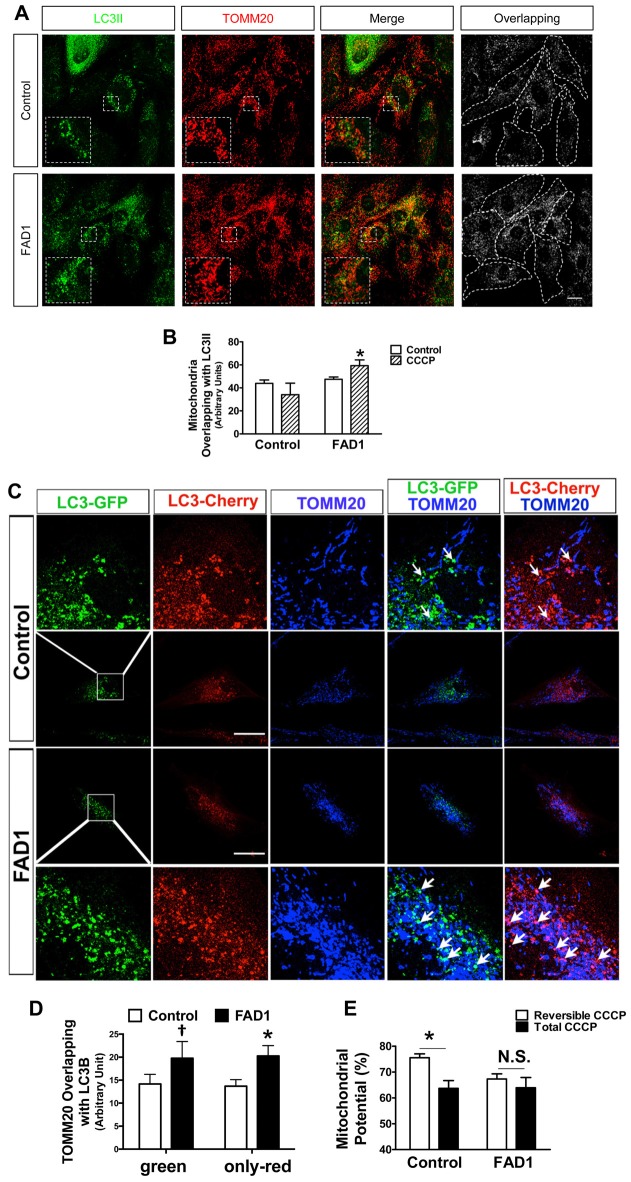
Mitochondria accumulation in autophagic vesicles (AV) and impaired mitochondrial potential recovery in FAD1 fibroblasts. **(A)** Representative confocal microscopy immunofluorescence images showing LC3 in green and TOMM20 in red of control and FAD1 fibroblasts treated with CCCP (20 μM) for 24 h. On the right, binary image representing the colocalization of both labels and dotted line delimits cytoplasm of each cell according to phalloidin staining (not shown). **(B)** Quantification of the colocalization between LC3 and TOMM20 expressed as area occupied by the overlapping elements per cell. **(C)** Representative confocal immunofluorescence images of fibroblasts infected with a lentivector encoding mCherry-EGFP-LC3B and treated with CCCP for 6 h showing TOMM20 in blue. The upper and lower rows show magnifications of the squared area. Arrows point colocalization areas. **(D)** Quantification of the colocalization between TOMM2 and green vesicles or only-red vesicles. **(E)** Mitochondrial membrane potential of the same fibroblasts treated with CCCP (20 μM) for 6 h and then allowed to recover for 1 h (Reversible CCCP) or treated for 7 h with CCCP (Total CCCP). Results are expressed as percentage with respect to the mean of untreated cells (*n* = 3 independent samples; Control/AD fibroblasts couples used AG12988/AG06848 **A,B,E** and AG04148/AG06840 **C,D**; ^†^*p* < 0.07; *p < 0.05). Scale bar: 40 μm.

Once a defect in autophagy had been corroborated in our FAD1-fibroblasts, we investigated if the proteins implicated in mitochondrial recycling process could be deregulated by the presence of the FAD1 *PSEN1* mutation. FAD1-fibroblasts showed an abnormal increased PARK2 (Parkin) levels compared with control samples in both couples (Figure 3A and Supplementary Figure S4A). On the other hand, Western blot analysis displayed that both FL and Δ1 isoforms of PINK1 were slightly decreased in *PSEN1*-FAD cells compared to controls after the treatment with CCCP for 24 h (Figure 3B). Similar diminished Δ1 PINK1 was observed in the second FAD1 fibroblasts (Supplementary Figure S4B). Additionally, we studied the recruitment of PARK2 to the mitochondria by immunofluorescence after CCCP insult for 1 h (Figures 3C,D). We observed that mitochondrial localization of PARK2 was increased in FAD1-fibroblasts suggesting an accumulation of damaged mitochondria that cannot be recycled by mitophagy due to the degradation phase deficiency described above. In fact, the analysis of mitochondrial surface (as measured by IF-labeling of the mitochondrial constitutively expressed protein TOMM20) of individual cells revealed increased mitochondrial content in FAD1 cells respective to controls (Figures 3E,F). Accordingly, we could observe an accumulation of mitochondria in AV after CCCP treatment measured by the increased colocalization of TOMM20 and LC3II (Figures 4A,B) suggesting a mitophagy impairment as a consequence of lysosome dysfunction. To further characterize the defect in mitophagy, we infected fibroblasts with a lentivector encoding mCherry-EGFP-LC3B, a tool that allows us to study the maturation of AV (Hansen and Johansen, 2011) as well as the colocalization of mitochondria with AV after the treatment with CCCP. An accumulation of mitochondria in autophagosomes (green and red fluorescence) could be observed in FAD1 cells compared to control cells (Figures 4C,D). Fusion of these autophagosomes with late endosomes to form amphisomes or to lysosomes to form autolysosomes destroys green fluorescence but remaining red fluorescence until the proteins are degraded in the autolysosome. In these vesicles with only red fluorescence, mitochondria and the rest of vesicle cargo should be rapidly digested and hardly detected. However, in FAD1 fibroblasts mitochondria were significantly accumulated in red-only amphisomes and autolysosomes, suggesting that their degradation might be impaired (Figures 4C,D). Finally, we studied mitochondrial membrane potential recovery using the probe DiOC6 (Sauvat et al., 2015) after a reversible challenge with CCCP. We observed a diminished membrane potential recovery after a reversible treatment with CCCP in FAD1-fibroblasts relative to controls, indicating a defect in mitochondrial recycling by mitophagy (Figure 4E). Therefore, these results indicate an impairment of the clearance of mitochondria by mitophagy.

### Autophagy Failure in *PSEN1*-FAD iPSC-Derived Neurons

**Figure 5:**
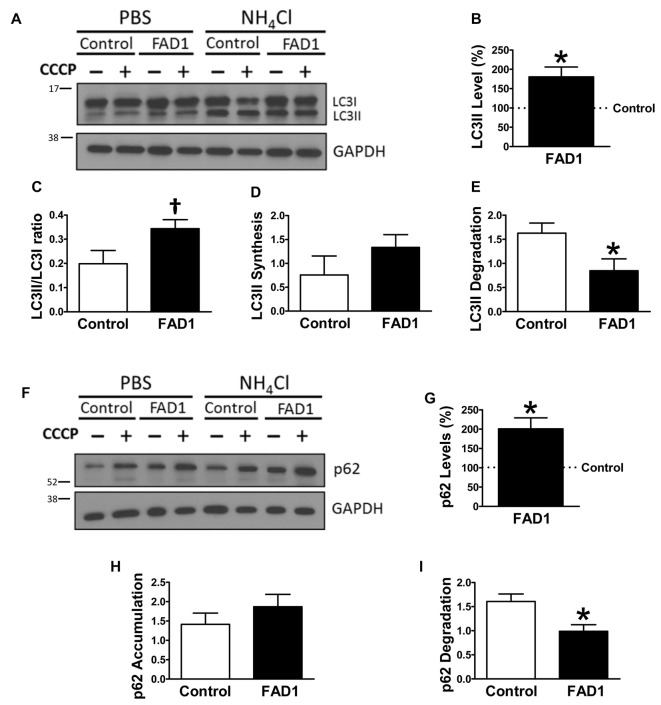
Impaired degradation phase of autophagy in FAD1-derived neurons. **(A)** Representative Western blot of LC3 expression in control and FAD1 neurons treated or not with CCCP (20 μM) in the absence or presence of NH_4_Cl (15 mM). **(B,C)** Quantification of LC3II levels **(B)** and LC3II/LC3I ratio **(C)** in FAD1 cells with respect to the control ones under basal conditions. **(D,E)** Quantification of LC3II synthesis **(D)** and degradation **(E)** ratios as described in “Materials and Methods” Section and Supplementary Figure S1. **(F,G)** Western blot of p62 expression after the treatment as in **(A)** and quantification of basal levels **(G)**. **(H,I)** Quantification of p62 synthesis **(H)** and degradation **(I)** ratios as described before (*n* = 3 independent experiments using neurons derived from the control and AD iPSC lines 6842 A/7671 C; ^†^*p* < 0.08; *p < 0.05).

**Figure 6:**
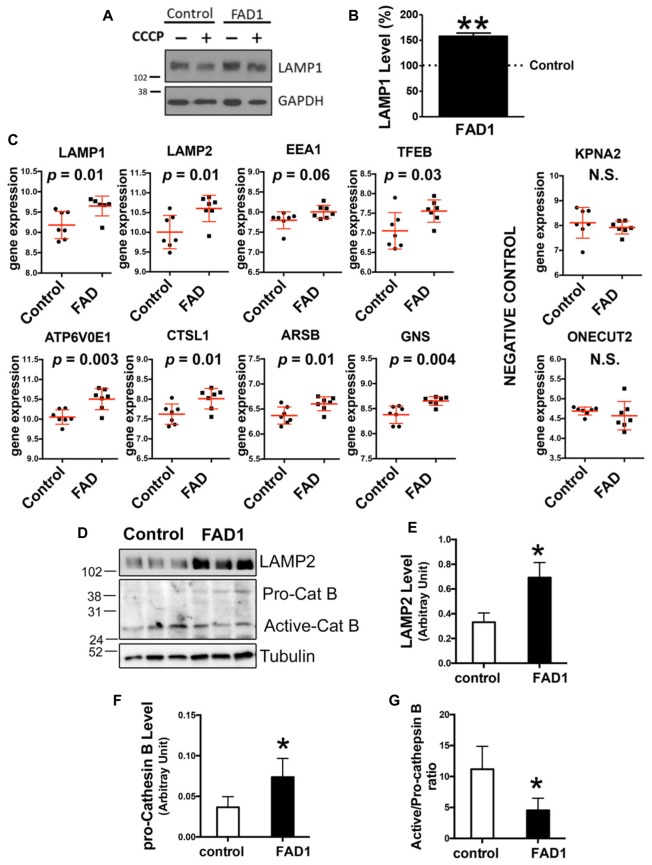
Lysosomal anomalies in FAD neurons. **(A,B)** Representative Western blot and quantification of LAMP1 protein levels **(B)** under basal conditions FAD1 iPSC-derived neurons with respect to controls. **(C)** Analysis of microarray expression of indicated genes. Graphs show dot plots of the expression of the genes using *n* = 7 controls and *n* = 7 FAD brain samples retrieved from the Antonell dataset (Antonell et al., 2013). Correspondent *p* value was determined by Student’s *t*-test and it is showed for each graph. **(D–G)** Representative Western blot of LAMP2 and cathepsin B of iPSC-derived neurons under basal conditions and quantification of LAMP2 levels **(E)**, pro-cathepsin B levels **(F)** and active-/pro-cathepsin B ratio **(G)** (*n* = 3 for **A,B,E** and *n* = 4 for **F,G** independent experiments using neurons derived from the control and AD iPSC lines 6842 A/7671 C; *p < 0.05; **p < 0.01).

Taking into account our previous results in FAD1 fibroblasts, we asked whether autophagy was impaired in human cortical neurons that contain an endogenous *PSEN1* mutation. With this aim we used iPSCs derived from fibroblasts from control and FAD1 patients generated by retroviral transduction of the canonical Yamanaka factors Oct4, KLF4, SOX2 and c-Myc as previously established (Sproul et al., 2014). We confirmed the expression of canonical pluripotency genes by immunofluorescence and qPCR analysis of iPSC colonies (Supplementary Figures S5A,B, respectively). Additionally, we corroborated the expression of specific markers from the three germ layers in spontaneously differentiated embryonic bodies validating their pluripotency (Supplementary Figure S5C). Cultures from iPSC lines were differentiated to neurons as previously described (Prè et al., 2014; Sproul et al., 2014). After 40 days of differentiation, control and FAD1 derived neurons were positive for mature neuron markers (Supplementary Figure S6A). On the other hand, FAD1 derived neurons exhibited higher levels of APP and similar levels of Tau compared with control ones (Supplementary Figure S6B). However, in the presence of CCCP, increased differences were observed in both APP and Tau, pointing out the relevance of mitophagy process in these AD related hallmarks (Supplementary Figures S6C–E). To functionally study autophagy, control and FAD1 iPSC-derived neurons were treated with CCCP for 24 h followed by an additional treatment of NH_4_Cl for 6 h in the presence of CCCP (Figure 5). Similar to what we showed for fibroblasts, FAD1 neurons exhibited an increased amount of autophagic vacuoles due to the higher levels of LC3II and LC3II/LC3I ratio under basal conditions (Figures 5A–C). These results correlate with slightly increased synthesis and markedly decreased degradation of AV observed in FAD1 neurons (Figures 5D,E). Parallel to this inhibition of degradation phase of autophagy, we found a significant increase of p62 levels in FAD1 neurons with respect to control neurons under basal conditions (Figures 5F,G) together with reduced p62 degradation ratio (Figure 5I). Degradation phase failure strongly suggest lysosomal dysfunction in FAD1 iPSC-derived neurons, however when we studied the levels of a lysosomal constitutive marker such as LAMP1 we observed dramatic increase of lysosome levels (Figures 6A,B). Trying to explain this augmented lysosomal content we performed an analysis of several lysosomal markers in a microarray database of brain samples from healthy (*n* = 7) and FAD patients (*n* = 7) associated to *PSEN1* mutations (Antonell et al., 2013). We could observe a significant increase of lysosomal markers such as LAMP1 and LAMP2 as well as the early endosomal marker EEA1 in FAD patients (Figure 6C). Accordingly, there was an increase of the master regulator of lysosomal biogenesis TFEB as well as other genes regulated by this factor such as ATP6V0E1, CTSL1, ARSB and GNS, but not in other unrelated genes such as KPNA2 or ONECT2 (Sardiello et al., 2009; Figure 6C). To explain why increased lysosomal biogenesis and lysosomal content correlated with lysosomal function impairment, we studied the processing of Cathepsin B that depends on lysosomal function in iPSC-derived neurons (Figures 6D–G). Although FAD1 neurons exhibited increased levels of pro-cathepsin B (Figure 6F), as expected from augmented lysosomal content measured by LAMP2 (Figure 6E), the activation ratio is significantly decrease suggesting impaired lysosomal function (Figure 6G).

### Misregulation of the Proteins Involved in Mitophagy in FAD1 iPSC-Derived Neurons

**Figure 7:**
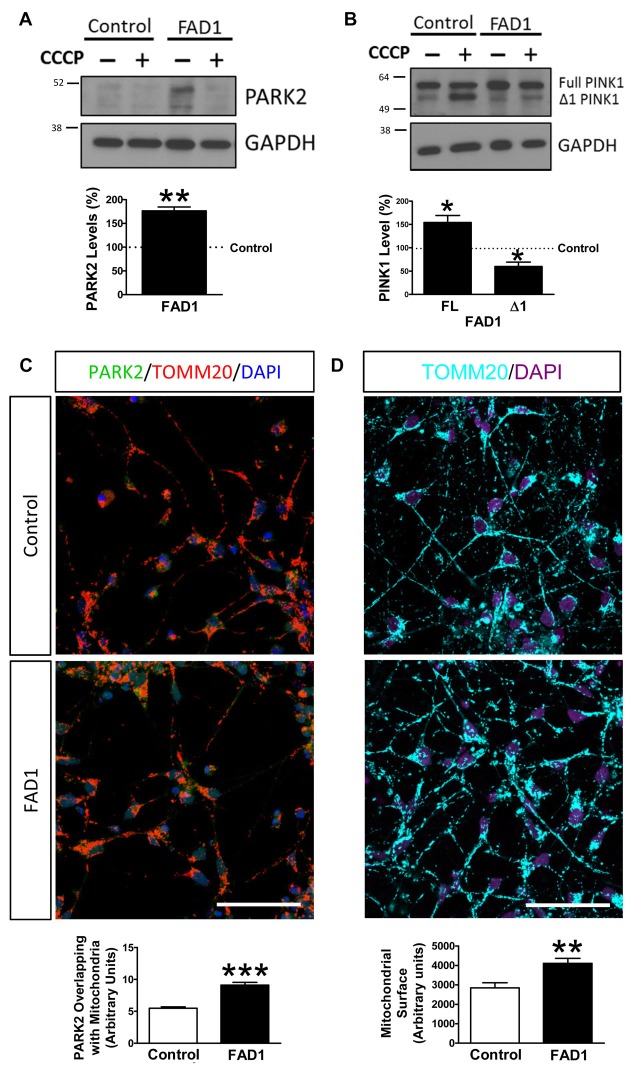
Mitophagy impairment in FAD1-derived neurons. **(A)** Representative Western blot of control and FAD1 neurons in the absence or presence of CCCP (20 μM) for 24 h and quantification of PARK2 levels under basal conditions. **(B)** Representative Western blot and quantification of FL-PINK1 and Δ1-PINK1 after the treatment with CCCP. **(C)** Representative confocal microscopy immunofluorescence images showing PARK2 in green, TOMM20 in red and DAPI in blue of control and FAD1 neurons treated with CCCP (20 μM) for 1 h. Quantification of the colocalization between PARK2 and TOMM20 expressed as area occupied by the overlapping elements per cell. **(D)** Representative confocal images of control and FAD1 neurons showing TOMM20 in cyan and DAPI in purple in basal conditions. Quantification of the mitochondrial surface per cell (*n* = 3 independent experiments using neurons derived from the control and AD iPSC lines 6842 A/7671 C, *p < 0.05; **p < 0.01; ***p < 0.001). Scale bar: 50 μm.

**Figure 8:**
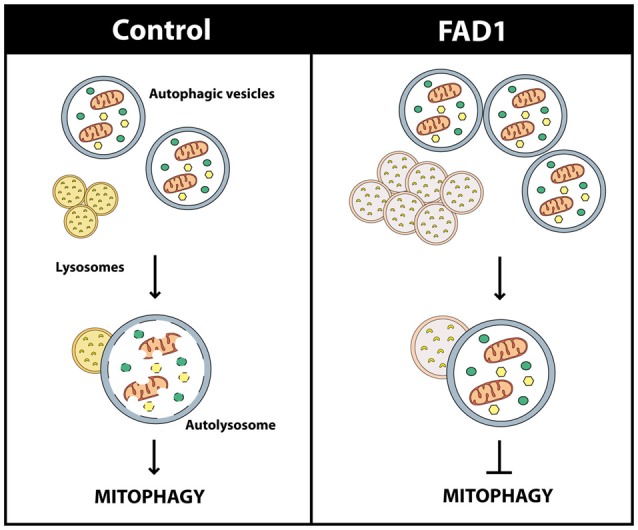
Scheme of the mitophagy failure in FAD1. Dysfunctional oversized lysosomes in FAD1 cells cause the accumulation of AV containing mitochondria showing that their recycling is impaired.

Once FAD1 neurons demonstrated similar autophagy alterations and lysosomal function impairment to what was observed in fibroblasts, we turned our attention to the proteins involved in mitophagy. Under basal conditions, FAD1 neurons revealed increased levels of PARK2 compared to control neurons (Figure 7A). Surprisingly, FAD1 neurons exhibited elevated levels of FL-PINK1 in contrast to Δ1-PINK1 isoform, which were diminished after the treatment with CCCP for 24 h (Figure 7B). In consonance, immunostaining displayed an elevated PARK2 localization on mitochondria in FAD1 neurons after 1 h treatment with CCCP, similar to the situation founded in FAD1 fibroblasts (Figure 7C). This result was accompanied by markedly increased mitochondrial surface in FAD1 neurons respect to control neurons (Figure 7D). All these result together suggest that, after CCCP treatment, FL-PINK1 was stabilized in dysfunctional mitochondria to where PARK2 was correctly recruited but, due to the previously demonstrated defect in the degradation phase of autophagy as a consequence of lysosome dysfunction, the mitochondria were not able to be recycled, thus causing the observed accumulation (See model in Figure 8).

## Discussion

Presenilins are ubiquitously expressed in many human tissues, including brain, heart, kidney and muscle (Lee et al., 1996). Moreover, high levels of *PSEN1* mRNA in both skin fibroblasts and brains from AD patients has been reported (Ikeda et al., 2000). FAD models allow characterization of pathology using a defined specific mutation and the development of *PSEN1* iPSCs is a useful tool to seek for potential alterations in FAD neurons. With this aim, we have focused our research in *PSEN1* A246E mutation since it is a well-characterized FAD mutation that shows typical phenotypes of AD with complete penetrance (Sherrington et al., 1995; Cruts et al., 1998).

We have used functional assays to analyze the mitochondrial recycling process in cell lines from FAD1 patients. The purpose was to compare the results obtained in peripheral cells with a human neural model generated from iPSC harboring the same FAD1 mutation. We have demonstrated a deregulation of autophagy as a result of a reduction of degradation phase in both FAD1 fibroblasts and iPSC-derived neurons. This may be caused by an impaired fusion of the AV with lysosomes or an inhibition of lysosomal function. Consistently, we could observe abnormal increased size of early endosomes and lysosomes correlating with diminished acidification of lysosomes in FAD1 fibroblasts in aggrement with the lysosome acidification deficiency reported by Lee et al. (2010). In the mentioned work, PSEN1 was demonstrated to act as chaperone protein that facilitates the N-glycosylation of V-ATPase subunit V0a1, which help V-ATPase traffic to lysosome and complete lysosome acidification. Accordingly, it has been demonstrated that the reacidification of lysosomes by cyclic adenosine monophosphate treatment was able to reverse observed LC3II accumulation observed in FAD1 fibroblasts (Coffey et al., 2014). Additionally, we have seen deficient lysosomal function in iPSC-derived neurons despite their increased lysosomal content. Furthermore, the increase of AV has been observed in dystrophic neurites around amyloid plaques (Sanchez-Varo et al., 2012). Likewise, the accumulation of p62 has been observed in neuronal and glial ubiquitin-containing inclusions (Kuusisto et al., 2001) as well as in neurofibrillary tangles (Kuusisto et al., 2002) in AD brain. Therefore, all these results together demonstrate that *PSEN1* mutations impair degradative phase of autophagy.

The study of mitophagy after a mitochondrial insult indicates that this process is severely affected due to the mitochondrial accumulation exhibited in both fibroblasts and iPSC-derived neurons from FAD1 patients. These two cell types showed a marked accumulation of PARK2 in mitochondria suggesting a correct PINK1-PARK2 labeling of depolarized mitochondria but, due to the degradation phase deficiency described before, there is an accumulation of damaged mitochondria that are unable to be recycled by mitophagy. This data correlates with previous results in 5xFAD mice that exhibited drastically increased PARK2 translocation to synaptosomal mitochondria in an age- dependent manner (Wang et al., 2016). Moreover, PARK2 accumulation could be due to proteasome degradation system saturation as a result of the autophagy impairment (Yamano et al., 2016). Noteworthy, we could observe an accumulation of AV containing mitochondria in our FAD1 fibroblasts, which correlates with what was previously reported in pyramidal neurons from AD patients suggesting a mitophagy alteration (Moreira et al., 2007a,b). Additionally, we have demonstrated a functional impairment in the mitochondrial membrane potential recovery in FAD1 fibroblasts. In FAD1 neurons we could observe a clear accumulation of PARK2 and FL-PINK1 what has been previously related with the accumulation of depolarized mitochondria unable to be degraded by autophagy (Narendra et al., 2010; Tanaka, 2010). The drastic accumulation of PARK2 in mitochondria together with the strong increased mitochondrial content in FAD1-iPSCs derived neurons suggests enhanced mitophagy failure in the neuronal model as compared to the fibroblast model. The critical dependence of neurons on mitochondrial function as well as the importance of autophagy in both normal neuronal function and neurodegeneration may explain the aggravation of the mitophagy failure observed in FAD1 neurons. Similar accumulation of PARK2 in mitochondria, increased LC3II levels and AV containing altered mitochondrial structures has been found in hippocampus of AD patients (Ye et al., 2015) confirming the relevance of our findings in FAD1 fibroblasts and iPSC derived neurons.

It would be interesting to study the dependence of the observed mitophagy deficiency on the accumulation of Aβ. To this aim, inhibitors of γ-secretase such as DAPT (Dovey et al., 2001) or BACE inhibitors (Baxter et al., 2007) would contribute to determine the dependence on Aβ production. However, due to the fact that the main differences between control and FAD1 fibroblast and iPSC derived neurons are in the Aβ_42_/Aβ_40_ ratio rather than in the total levels of Aβ, which have been found similar (Sproul et al., 2014), it is probable that described mitophagy failure may be independent of Aβ levels. Although Aβ_42_ contribution cannot be ruled out so far, previous work demonstrating the role of PSEN1 in lysosomal acidification (Lee et al., 2010) together with the degradation phase impairment that we have observed led us to conclude that the phenotype may be related to a loss of function rather than a gain of toxic function.

In summary, our findings indicate that both skin fibroblasts and iPSC-derived neurons from FAD1 patients harboring *PSEN1* A246E mutation demonstrate a marked mitophagy failure as a consequence of lysosomal dysfunction being exacerbated in the neuronal model. We have previously described similar mitophagy failure in fibroblasts and patients’ brain samples in sporadic AD (Martín-Maestro et al., 2016), although the etiology of the defect is different. In sporadic cases, the deficit is due to insufficient labeling of mitochondria to be degraded by mitophagy; while, in familial cases, mitochondria are correctly tagged for recycling but there is a problem in the degradation phase of autophagy. Therefore, mitochondrial recycling problem can be considered a common feature of both sporadic and familiar cases of the disease, highlighting the relevance of the finding. We demonstrated a mitochondrial recycling deficit in a neuronal model derived from patients. The present work also demonstrates that FAD1-fibroblasts retain mitophagy pathology found in FAD1-neurons and patient brains, further validating their use as cell models for the study of AD.

## Author Contributions

PM-M and VG-E: conception and design of the work, acquisition, analysis and interpretation of data, manuscript writing, critically revising the work for important intellectual content, final approval of the version to be published. RG: acquisition, analysis and interpretation of data, critically revising the work for important intellectual content, final approval of the version to be published. EG: acquisition and analysis of the data, final approval of the version to be published. AAS, SN and OA: design of the work, interpretation of data, critically revising the work for important intellectual content, final approval of the version to be published. LCA developed mCherry-EGFP-LC3B lentiviral vector, final approval of the version to be published. JA: conception and design of the work, interpretation of data, manuscript writing, critically revising the work for important intellectual content, final approval of the version to be published.

## Conflict of Interest Statement

The authors declare that the research was conducted in the absence of any commercial or financial relationships that could be construed as a potential conflict of interest.

## Abbreviations

Aβ: Amyloid-beta
AD: Alzheimer disease
APP: amyloid precursor protein
AV: autophagic vesicles
CCCP: carbonyl cyanide m-chlorophenylhydrazone
FAD: Familial Alzheimer’s disease
FAD1: Familial Alzheimer’s disease associated to presenilin 1 A246E mutation
GAPDH: Glyceraldehyde-3-phosphate dehydrogenase
LAMP1: Lysosomal-associated membrane protein 1
LAMP2: Lysosomal-associated membrane protein 2
LC3: Microtubule-associated protein 1 light chain 3
MAPT/Tau: microtubule-associated protein tau
ΔΨm: mitochondrial membrane potential
PARK2: Parkin RBR E3 ubiquitin protein ligase
PINK1: PTEN-induced putative kinase 1
PSEN: presenilin
TOMM20: Translocase of outer mitochondrial membrane 20 homolog.
