# Supplementary material for: Mitophagy Failure in Fibroblasts and iPSC-Derived Neurons of Alzheimer’s Disease-Associated Presenilin 1 Mutation
Source: Front Mol Neurosci. 2017 Sep 14;10:291. doi: 10.3389/fnmol.2017.00291 (PMC5603661; doi:10.3389/fnmol.2017.00291)
Supplement: Supplementary file 1 [file Presentation_1.pdf]

Supplemental Figure 1

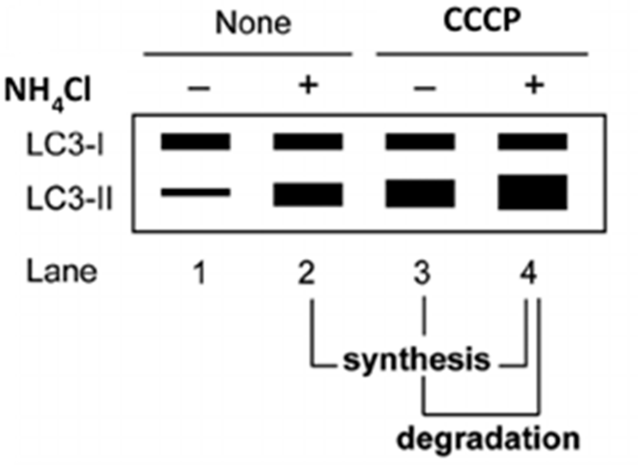

Figure S1: Scheme of the autophagy flux study. The autophagic synthesis quantification was calculated as the ratio between the condition were we induced autophagy by the treatment with CCCP and blocked lysosomal degradation by the treatment with NH_4Cl divided by the condition were cells were treated only with NH_4Cl (lane 4/lane 2). On the other hand, the autophagic degradation quantification was calculated as the relation between the condition were we induced autophagy by the treatment with CCCP and blocked lysosomal degradation by the treatment with NH_4Cl divided by the condition were autophagy was induced by CCCP treatment (lane 4/lane 3). Modified from Figure 1C of *Rubinsztein et al. (2009) In search of an "autophagomometer". Autophagy. Jul;5:585-589*

Supplemental Figure 2

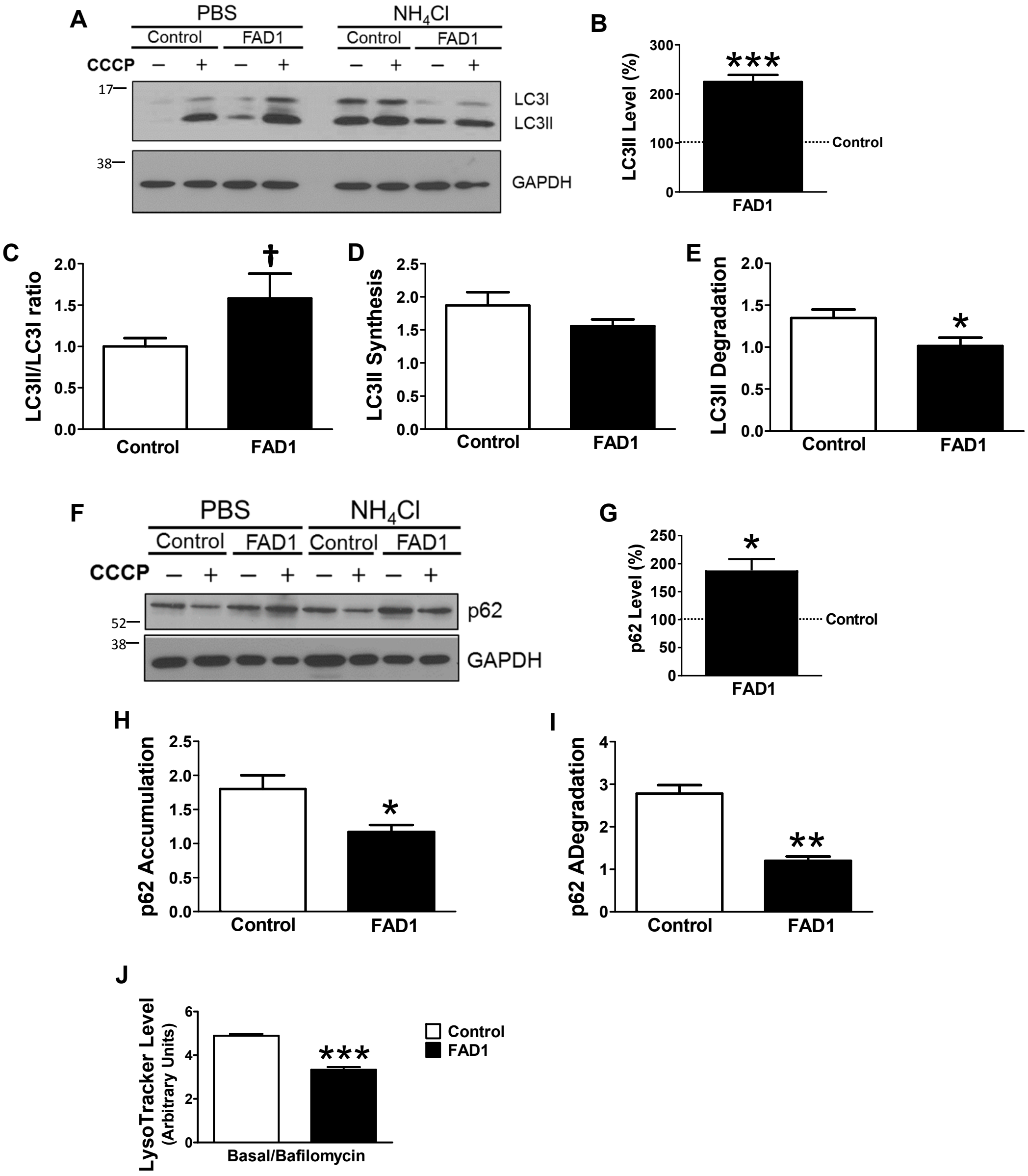

Figure S2. Autophagy degradation phase impairment in other line of FAD1 fibroblasts. (A) Representative Western blot of LC3 expression for the study of autophagy flux as explained in Experimental Procedures in control and FAD1 fibroblasts treated or not with CCCP (20 µM) in the absence or presence of NH_4Cl (15 mM). (B-C) Quantification of LC3II levels (B) and LC3II/LC3I ratio (C) in FAD1 cells with respect to the control ones under basal conditions. (D-E) Quantification of LC3II synthesis (D) and degradation (E) ratios as described in Experimental Procedures. (F-G) Western blot of p62 expression after the treatment as in (A) and quantification of basal levels (G). (H-I) Quantification of p62 accumulation (H) and degradation (I) ratios. (J) Quantification of the lysosomal acidity represented by the ratio between untreated and bafilomycin (100 nM) treated cells using Lysotracker probe by flow cytometry. (n=3 independent experiments using the control/AD fibroblast couple AG12988/AG06844; †p< 0.08; *p<0.05; **p<0.01; ***p<0.001).

Supplemental Figure 3

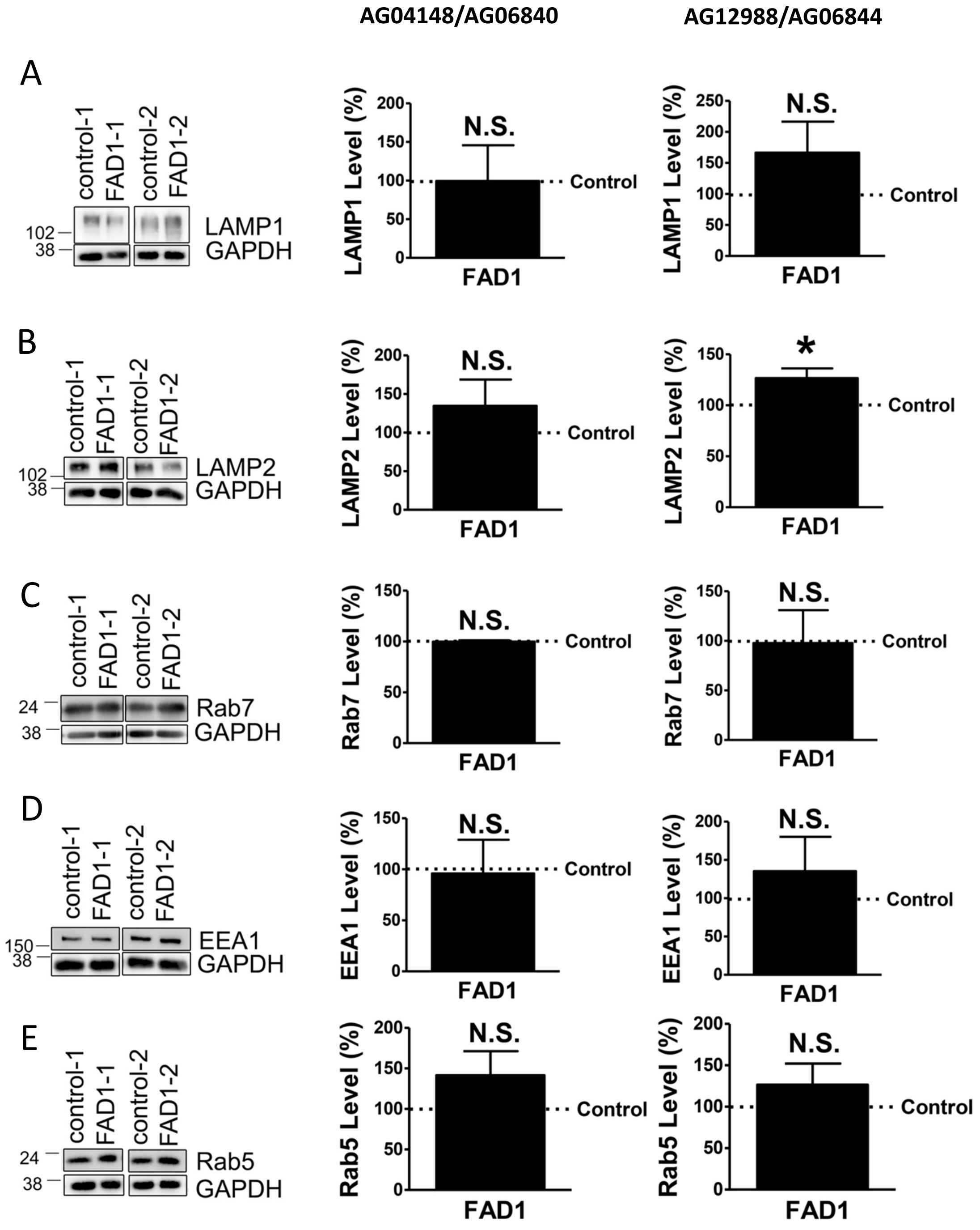

Figure S3. Vesicles markers in FAD1 fibroblasts. Representative Western blot of the following markers: LAMP1 (A), LAMP2 (B), Rab7 (C), EEA1 (D) and Rab5 (E), as well as quantification of the data in fibroblasts control/FAD1 couple 1 AG04148/AG06840 and couple 2 AG12988/AG06844. Graphs represent means and standard deviations n=3 independent samples except for LAMP1 AG04148/AG06840 where n=6. **p*<0.05; N.S.= not significant.

Supplemental Figure 4

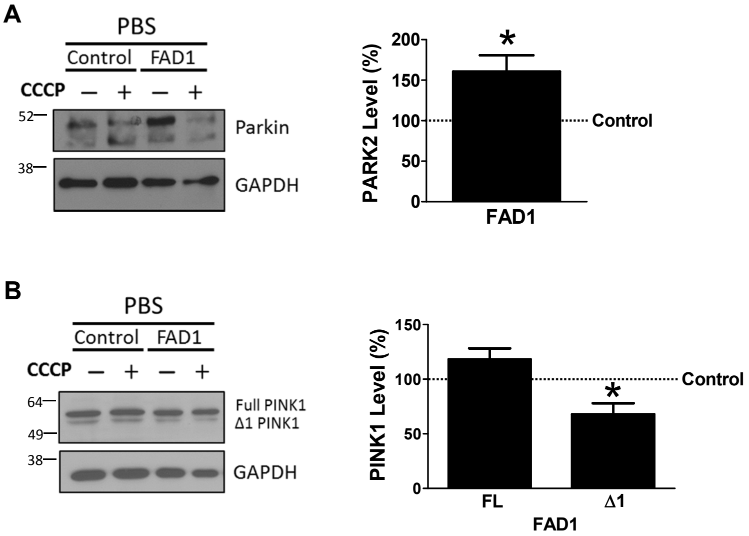

Figure S4. Dysfunctional mitochondria clearance in FAD1 fibroblasts.
(A) Representative Western blot of control and FAD1 fibroblasts in the absence or presence of CCCP (20 μM) for 24 hrs and quantification of PARK2 levels under basal conditions. (B) Representative Western Blot and quantification of FL-PINK1 and Δ1-PINK1 after the treatment with CCCP. (n=3 independent experiments using the control/AD fibroblast couple AG12988/AG06844; *p<0.05).

Supplemental Figure 5

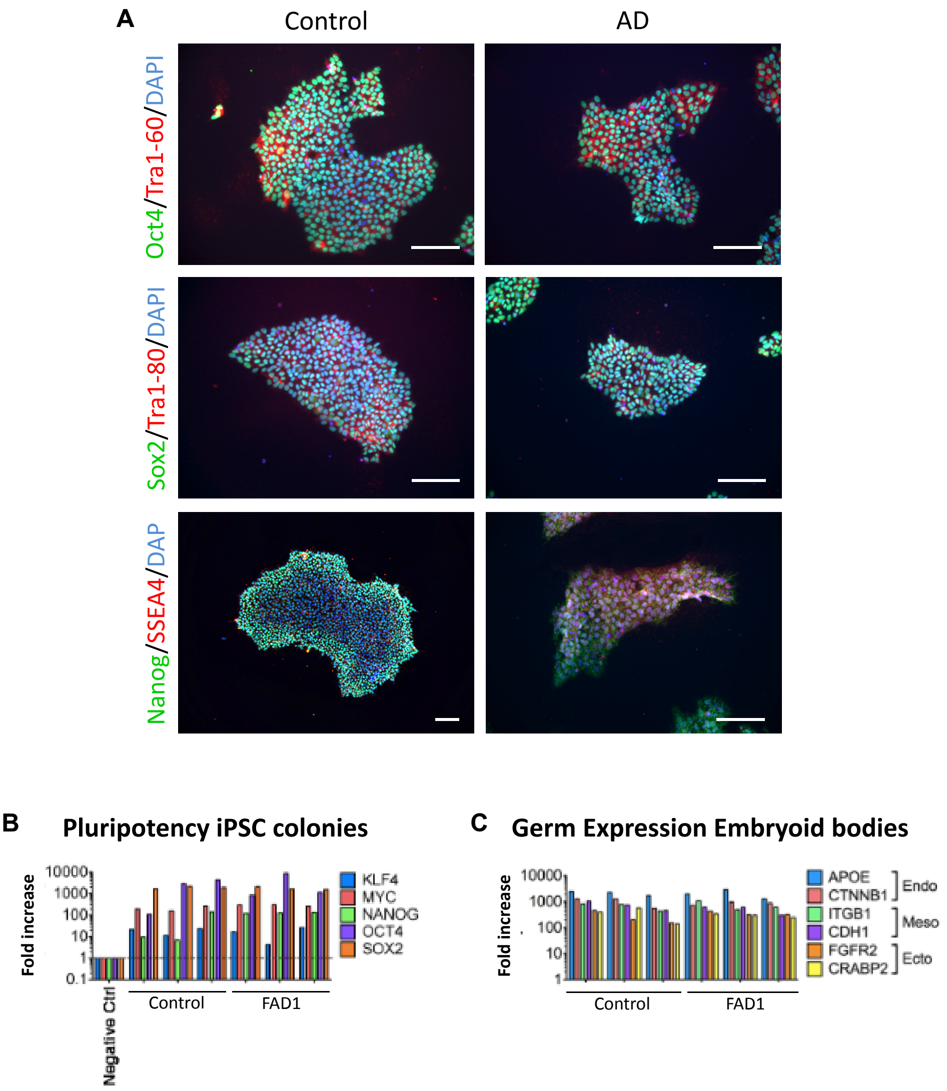

Figure S5. Characterization of pluripotency markers of control and FAD1 iPSC colonies. (**A**) Immunofluorescence representative images of stablished iPSCs from both control and FAD1 patients showing pluripotent stem cell markers Oct4/Tra1-60, Sox2/Tra1-81, NANOG/SSEA-4 and DAPI for nuclei staining. Scale bar: 50 µm. (**B**) RNA expression of detailed canonical pluripotency genes in control and FAD1 lines with respect to fibroblasts (Negative Ctrl). (**C**) RNA expression of specified canonical germ layer markers in spontaneously differentiated embryoid bodies relative to control iPSCs (not shown). Graphs show the median *t*-score of the expression levels of n=3.

Supplemental Figure 6

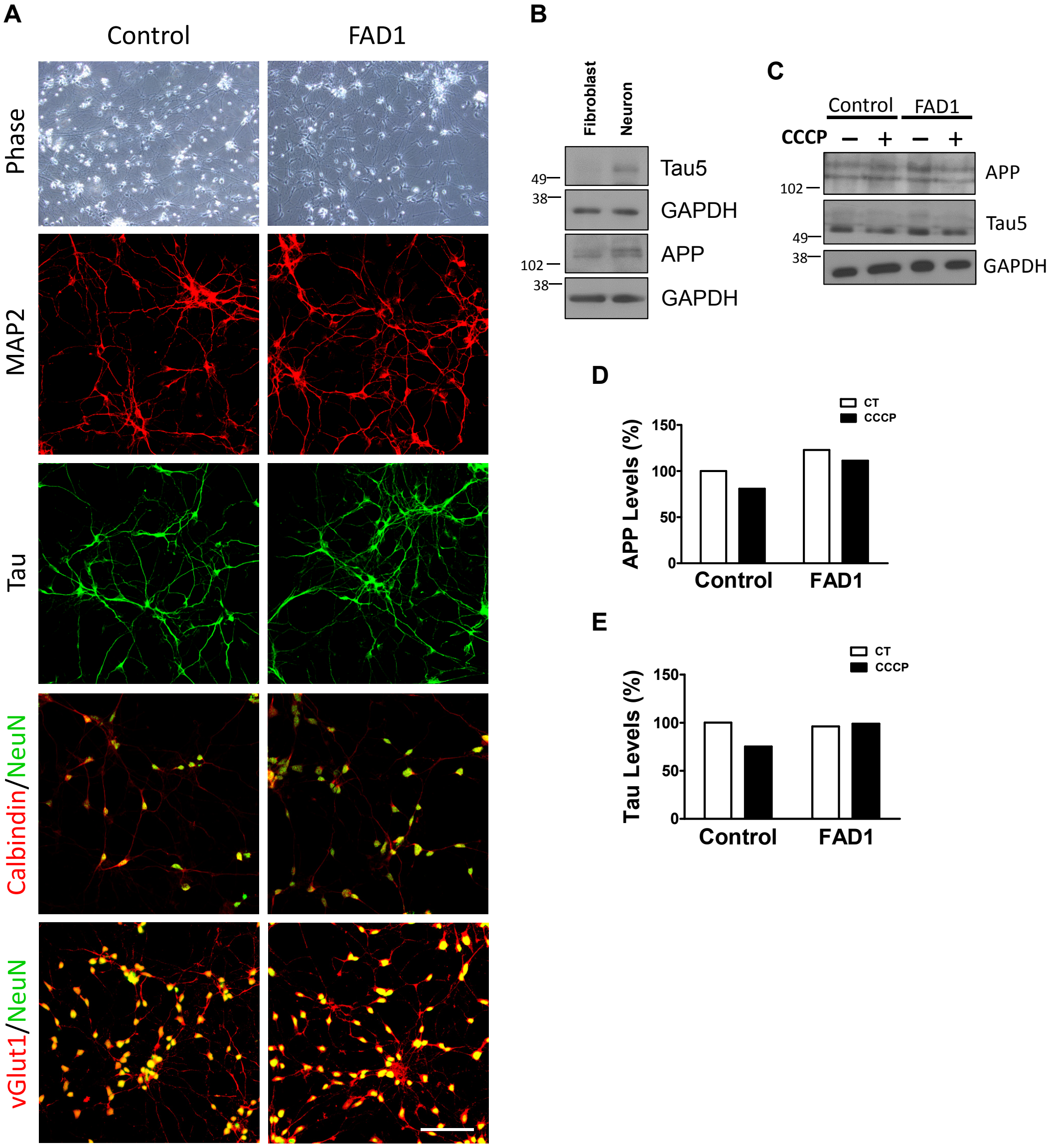

Figure S6. Characterization of control and FAD1-derived neurons. (A) Control and FAD1 lines were differentiated to neurons for 40 days and immunostained with general neuronal markers MAP2, Tau, NeuN, Calbindin or synaptic markers such as vGlut1. Scale bar: 200 µm. (B) Representative Western blot of the proteins involved in AD pathology in FAD1 fibroblasts and iPSC-derived neurons under basal conditions. (C) Representative Western blot of APP and Tau in control- and FAD1-derived neurons in the absence or presence of CCCP (20 µM) besides the (D) quantification of APP and (E) total Tau levels with respect to untreated control neurons.
